# Editorial: Neuro-immune-endocrine mechanism of acupuncture in the treatment for nervous system diseases

**DOI:** 10.3389/fneur.2024.1449040

**Published:** 2024-07-26

**Authors:** Xiaowen Cai, Huacong Liu, Zhang-Jin Zhang, Chunzhi Tang, Yong Huang

**Affiliations:** ^1^The Tenth Affiliated Hospital, Southern Medical University (Dongguan People's Hospital), Dongguan, Guangdong, China; ^2^School of Traditional Chinese Medicine, Southern Medical University, Guangzhou, Guangdong, China; ^3^School of Chinese Medicine, The University of Hong Kong, Pokfulam, Hong Kong SAR, China; ^4^Medical College of Acu-Moxi and Rehabilitation, Guangzhou University of Chinese Medicine, Guangzhou, Guangdong, China

**Keywords:** acupuncture, neuro-immune-endocrine, nervous system diseases, mechanisms, ischemic stroke

Nervous system diseases, such as cerebrovascular diseases, spinal cord diseases, and neurodegenerative diseases, are characterized by a high incidence rate, high mortality, and intractability. The pathogenesis of nervous system diseases is complex, with key features including neurodegeneration and neuroinflammation. Recent research has increasingly emphasized the significant role of the neuro-immune-endocrine mechanism in the development of neurological diseases. Neuroimmune endocrine dysfunction is a significant mechanism in the pathogenesis of various nervous system diseases, including stroke, Alzheimer's disease, Parkinson's disease, multiple sclerosis, and depression. Additionally, neuroendocrine abnormalities are implicated in affective disorders, with the hypothalamic-pituitary-adrenal axis playing a critical role in depressive symptoms and recurrence of depression (1). Researchers are continuously investigating neurological disorders and actively pursuing innovative and efficacious treatment modalities. Acupuncture, a traditional therapy with a history of treating nervous system ailments like stroke and depression for millennia, has garnered widespread recognition for its safety and efficacy (2). In recent years, there has been a growing interest in the potential of acupuncture to modulate the neuro-endocrine-immune system in the context of neurological disorders. It has been demonstrated that acupuncture stimulates the neuroendocrine system to release neurotransmitters and neuropeptides within the body (3). By releasing neurotransmitters, hormones, and other substances that interact with immune organs and cells, the neuroendocrine system indirectly affects the immune system. Nevertheless, additional research is needed to fully understand the regulatory mechanisms of acupuncture on the nervous-immune-endocrine systems. As such, the primary objective of this Research Topic is to investigate and provide further evidence on the neuroimmune endocrine mechanisms underlying acupuncture treatment for nervous system diseases.

The Research Topic included four articles related to “*Neuro-immune-endocrine mechanism of acupuncture in the treatment for nervous system diseases*”.

The immune response plays an important role in the pathogenesis of ischemic stroke (4). Kuang et al. conducted a comprehensive review of the immune responses involving immune cell, cytokines, and immune organ following ischemic stroke. Based on a substantial literature review, this study analyzed acupuncture's immunoregulation effects on the central nervous system, peripheral immunity, and factors that influence its effectiveness. The intricate process by which acupuncture treats ischemic stroke primarily involves restoring and maintaining the dynamic equilibrium of the immune system, rather than solely enhancing or inhibiting immune responses.

Yu et al. conducted a comprehensive examination of the effects of acupuncture stimulation on local inflammatory response, as well as the interactions between the nervous and immune systems, using pertinent animal studies. The researchers found that acupuncture modulates the immune response of various cell types, including endothelial cells, neutrophils, macrophages, fibroblasts, and mast cells, through local biological and mechanical alterations. Moreover, acupuncture has the potential to modulate systemic inflammatory responses by engaging the vagus nervous system. The interaction between the nervous and immune systems serves as a crucial mechanism through which acupuncture exerts its regulatory effects on inflammatory processes.

Acupuncture can effectively improve limb dysfunction after acute stroke. Wang et al. discussed the dose effect relationship between different acupuncture doses (frequency, needle retention time, course of treatment) and clinical efficacy. Research has found that the optimal frequency for acupuncture is low frequency (1 time/2 days) and moderate frequency (1 time/day), while the optimal course of acupuncture is short-term (< 14 days). The dose-response relationship between the optimal needle retention time and clinical efficacy remains to be confirmed through research.

Xingnao Kaiqiao Acupuncture, a technique developed by Academician Shi Xuemin, is commonly utilized in the rehabilitation of stroke patients (5). Tian et al. explored the central mechanisms underlying the efficacy of Xingnao Kaiqiao acupuncture in addressing limb dysfunction following ischemic stroke using multimodal MRI technology. This research aims to enhance understanding of the cerebral mechanisms associated with the therapeutic effects of Xingnao Kaiqiao acupuncture.

In conclusion, this study delved into the significant discoveries made by researchers in the realm of acupuncture, specifically concerning the neuro-immune-endocrine mechanism of acupuncture in addressing neurological disorders. These findings offer valuable insights for researchers and healthcare practitioners seeking to enhance their understanding of the underlying mechanisms involved in utilizing acupuncture for the treatment of neurological conditions, thus facilitating the refinement of acupuncture's efficacy in managing a range of neurological disorders.

## Author contributions

XC: Writing – original draft. HL: Writing – original draft. Z-JZ: Writing – original draft. CT: Writing – review & editing. YH: Writing – review & editing.
